# 4,5-Diphenyl-2-*p*-tolyl-1*H*-imidazol-3-ium perchlorate

**DOI:** 10.1107/S1600536809024325

**Published:** 2009-07-04

**Authors:** Li Zhang

**Affiliations:** a Ordered Matter Science Research Center, College of Chemistry and Chemical Engineering, Southeast University, Nanjing 210096, People’s Republic of China

## Abstract

In the title compound, C_22_H_19_N_2_^+^·ClO_4_^−^, the three pendant aromatic rings are twisted from the plane of the imidazolium ring by dihedral angles of 17.3 (2), 65.7 (2) and 3.4 (2)°. In the crystal structure, N—H⋯O and N—H⋯(O,O) hydrogen bonds link the ions, forming a ribbon-like structure along the *a* axis.

## Related literature

For general background to imidazole derivatives, see: Fu & Xiong (2008 ▶); Huang *et al.* (2008 ▶). For applications of metal-organic coordination compounds, see: Fu *et al.* (2007 ▶, 2008 ▶); Huang *et al.* (1999 ▶); Liu *et al.* (1999 ▶); Xie *et al.* (2003 ▶); Zhang *et al.* (2000 ▶, 2001 ▶).
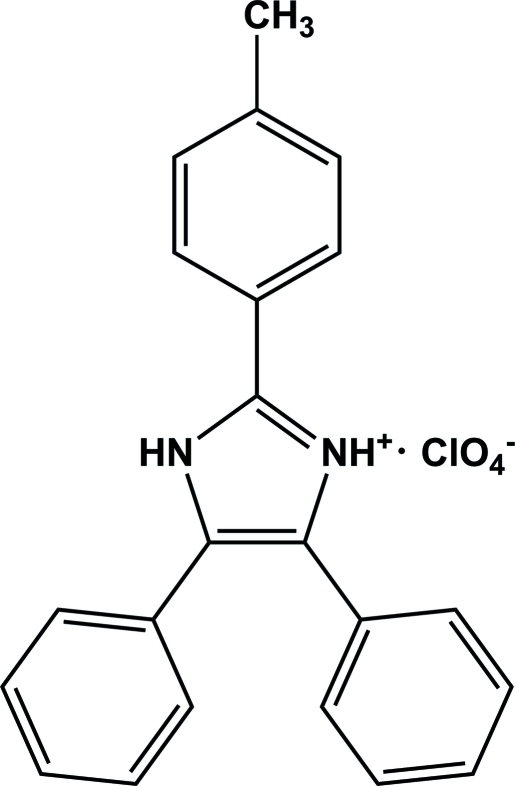


## Experimental

### 

#### Crystal data

C_22_H_19_N_2_^+^·ClO_4_^−^
*M* *_r_* = 410.84
Monoclinic, 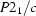

*a* = 9.1964 (18) Å
*b* = 9.921 (2) Å
*c* = 21.489 (4) Å
β = 94.16 (3)°
*V* = 1955.4 (7) Å^3^
*Z* = 4
Mo *K*α radiation
μ = 0.23 mm^−1^
*T* = 298 K
0.45 × 0.40 × 0.25 mm

#### Data collection

Rigaku Mercury2 diffractometer
Absorption correction: multi-scan (*CrystalClear*; Rigaku, 2005 ▶) *T*_min_ = 0.945, *T*_max_ = 1.000 (expected range = 0.893–0.945)
19242 measured reflections
4474 independent reflections
2602 reflections with *I* > 2σ(*I*)
*R*_int_ = 0.070

#### Refinement

*R*[*F* ^2^ > 2σ(*F* ^2^)] = 0.072
*wR*(*F* ^2^) = 0.214
*S* = 1.04
4474 reflections
271 parameters
24 restraints
H atoms treated by a mixture of independent and constrained refinement
Δρ_max_ = 0.54 e Å^−3^
Δρ_min_ = −0.35 e Å^−3^

### 

Data collection: *CrystalClear* (Rigaku, 2005 ▶); cell refinement: *CrystalClear*; data reduction: *CrystalClear*; program(s) used to solve structure: *SHELXS97* (Sheldrick, 2008 ▶); program(s) used to refine structure: *SHELXL97* (Sheldrick, 2008 ▶); molecular graphics: *SHELXTL* (Sheldrick, 2008 ▶); software used to prepare material for publication: *SHELXTL*.

## Supplementary Material

Crystal structure: contains datablocks I, global. DOI: 10.1107/S1600536809024325/ci2832sup1.cif

Structure factors: contains datablocks I. DOI: 10.1107/S1600536809024325/ci2832Isup2.hkl

Additional supplementary materials:  crystallographic information; 3D view; checkCIF report

## Figures and Tables

**Table 1:** Hydrogen-bond geometry (Å, °)

*D*—H⋯*A*	*D*—H	H⋯*A*	*D*⋯*A*	*D*—H⋯*A*
N1—H1⋯O3^i^	0.89 (4)	2.05 (4)	2.943 (4)	175 (3)
N2—H2⋯O1^ii^	0.81 (4)	2.33 (4)	3.037 (5)	146 (3)
N2—H2⋯O1^iii^	0.81 (4)	2.50 (4)	3.196 (5)	145 (3)

Symmetry codes: (i) 
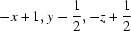
; (ii) 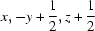; (iii) 
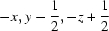
.

**Fig. 1.**
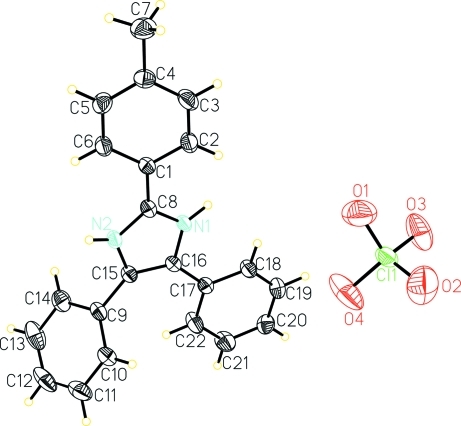
The molecular structure of the title compound, with the atomic numbering scheme. Displacement ellipsoids were drawn at the 30% probability level.

**Fig. 2.**
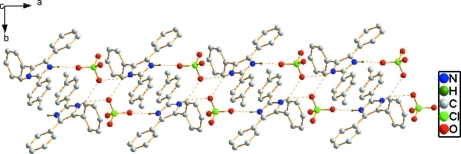
A hydrogen-bonded (dashed lines) ribbon in the title compound. H atoms not involved in hydrogen bonding have been omitted for clarity.

## supplementary crystallographic information

### Comment

The construction of metal-organic coordination compounds has attracted much attention owing to potential functions such as permittivity, fluorescence, magnetism and optical properties (Fu *et al.*, 2007, 2008; Huang *et al.*, 1999; Liu *et al.*, 1999; Xie *et al.*, 2003; Zhang *et al.*, 2000, 2001). Imidazole derivatives are a class of excellent ligands because of their multiple coordination modes as ligands to metal ions and for the construction of novel metal-organic frameworks (Huang *et al.* 2008; Fu & Xiong 2008). We report here the crystal structure of the title compound, 4,5-diphenyl-2-*p*-tolyl-1*H*-imidazolium perchlorate.

The title compound contains an organic cation and a ClO_4_^-^ anion in the asymmetric unit. The imidazole N atom in the 3-position is protonated. The C1-C6, C9-C14 and C17-C22 benzene rings form dihedral angles of 17.3 (2)°, 65.7 (2)° and 3.4 (2)°, respectively, with the imidazolium ring.

The crystal packing is stabilized by N—H···O hydrogen bonds which form a ribbon-like structure extending along the *a* axis (Table 1 and Fig. 2).

### Experimental

1,2-Diphenyl-ethane-1,2-dione (20 mmol), 4-methylbenzaldehyde (20 mmol) and amine acetate (50 mmol) were dissolved in 60 ml of HOAc under nitrogen protection. The mixture was stirred at 383 K for 20 h. The resulting solution was poured into ice water (200 ml) and after neutralizing the mixture with NaOH (6 mol/*L*) a white solid was obtained. The crude product was filtered and washed with distilled water. The crude product was dissolved in ethanol (150 ml)-perchloric acid (1 ml) and recrystallized to yield colourless block-shaped crystals of the title compound.

### Refinement

H atoms attached to N atoms were located in a difference Fourier map and refined freely. H atoms attached to C atoms were positioned geometrically and treated as riding, with C–H = 0.93 Å (aromatic) or 0.96 Å (methyl) and U_iso_(H) = 1.2-1.5U_eq_(C). The displacement parameters of O atoms were restrained to an approximate isotropic behaviour.

### Crystal data

**Table:**

C_22_H_19_N_2_^+^·ClO_4_^−^	*F*(000) = 856
*M**_r_* = 410.84	*D*_x_ = 1.396 Mg m^−^^3^
Monoclinic, *P*2_1_/*c*	Mo *K*α radiation, λ = 0.71073 Å
Hall symbol: -P 2ybc	Cell parameters from 2602 reflections
*a* = 9.1964 (18) Å	θ = 3.0–27.5°
*b* = 9.921 (2) Å	µ = 0.23 mm^−^^1^
*c* = 21.489 (4) Å	*T* = 298 K
β = 94.16 (3)°	Block, colourless
*V* = 1955.4 (7) Å^3^	0.45 × 0.40 × 0.25 mm
*Z* = 4	

### Data collection

**Table:**

Rigaku Mercury2 diffractometer	4474 independent reflections
Radiation source: fine-focus sealed tube	2602 reflections with *I* > 2σ(*I*)
graphite	*R*_int_ = 0.070
Detector resolution: 13.6612 pixels mm^-1^	θ_max_ = 27.5°, θ_min_ = 3.0°
CCD profile fitting scans	*h* = −11→11
Absorption correction: multi-scan (*CrystalClear*; Rigaku, 2005)	*k* = −12→12
*T*_min_ = 0.945, *T*_max_ = 1.000	*l* = −27→27
19242 measured reflections	

### Refinement

**Table:**

Refinement on *F*^2^	Primary atom site location: structure-invariant direct methods
Least-squares matrix: full	Secondary atom site location: difference Fourier map
*R*[*F*^2^ > 2σ(*F*^2^)] = 0.072	Hydrogen site location: inferred from neighbouring sites
*wR*(*F*^2^) = 0.214	H atoms treated by a mixture of independent and constrained refinement
*S* = 1.04	*w* = 1/[σ^2^(*F*_o_^2^) + (0.103*P*)^2^ + 0.7747*P*] where *P* = (*F*_o_^2^ + 2*F*_c_^2^)/3
4474 reflections	(Δ/σ)_max_ = 0.001
271 parameters	Δρ_max_ = 0.54 e Å^−^^3^
24 restraints	Δρ_min_ = −0.35 e Å^−^^3^

### Special details

**Table:**

Geometry. All e.s.d.'s (except the e.s.d. in the dihedral angle between two l.s. planes) are estimated using the full covariance matrix. The cell e.s.d.'s are taken into account individually in the estimation of e.s.d.'s in distances, angles and torsion angles; correlations between e.s.d.'s in cell parameters are only used when they are defined by crystal symmetry. An approximate (isotropic) treatment of cell e.s.d.'s is used for estimating e.s.d.'s involving l.s. planes.
Refinement. Refinement of *F*^2^ against ALL reflections. The weighted *R*-factor *wR* and goodness of fit *S* are based on *F*^2^, conventional *R*-factors *R* are based on *F*, with *F* set to zero for negative *F*^2^. The threshold expression of *F*^2^ > σ(*F*^2^) is used only for calculating *R*-factors(gt) *etc*. and is not relevant to the choice of reflections for refinement. *R*-factors based on *F*^2^ are statistically about twice as large as those based on *F*, and *R*- factors based on ALL data will be even larger.

### Fractional atomic coordinates and isotropic or equivalent isotropic displacement parameters (Å^2^)

**Table:**

	*x*	*y*	*z*	*U*_iso_*/*U*_eq_	
N1	0.4669 (3)	0.2183 (3)	0.48657 (13)	0.0395 (7)	
H1	0.549 (4)	0.216 (3)	0.4671 (16)	0.042 (9)*	
N2	0.2652 (3)	0.1423 (3)	0.51476 (13)	0.0438 (7)	
H2	0.186 (4)	0.106 (4)	0.5140 (16)	0.045 (11)*	
C1	0.3452 (3)	0.0484 (3)	0.41492 (15)	0.0396 (8)	
C2	0.4283 (4)	0.0746 (4)	0.36447 (17)	0.0570 (10)	
H2A	0.4916	0.1478	0.3658	0.068*	
C3	0.4170 (4)	−0.0072 (5)	0.31297 (18)	0.0643 (11)	
H3	0.4739	0.0112	0.2799	0.077*	
C4	0.3242 (4)	−0.1154 (4)	0.30873 (17)	0.0510 (9)	
C5	0.2414 (4)	−0.1400 (4)	0.35845 (18)	0.0563 (10)	
H5	0.1777	−0.2130	0.3565	0.068*	
C6	0.2499 (4)	−0.0604 (4)	0.41063 (17)	0.0505 (9)	
H6	0.1918	−0.0791	0.4432	0.061*	
C7	0.3166 (6)	−0.2061 (5)	0.2522 (2)	0.0741 (13)	
H7A	0.4118	−0.2416	0.2464	0.111*	
H7B	0.2827	−0.1554	0.2160	0.111*	
H7C	0.2505	−0.2791	0.2583	0.111*	
C8	0.3585 (3)	0.1336 (3)	0.47026 (15)	0.0408 (8)	
C9	0.2290 (4)	0.2630 (4)	0.61419 (16)	0.0452 (8)	
C10	0.1687 (4)	0.3869 (4)	0.62354 (19)	0.0593 (10)	
H10	0.1754	0.4537	0.5935	0.071*	
C11	0.0982 (5)	0.4148 (6)	0.6766 (2)	0.0774 (14)	
H11	0.0572	0.4993	0.6820	0.093*	
C12	0.0891 (5)	0.3171 (7)	0.7213 (2)	0.0850 (17)	
H12	0.0471	0.3368	0.7583	0.102*	
C13	0.1420 (6)	0.1908 (7)	0.7113 (2)	0.0880 (17)	
H13	0.1301	0.1232	0.7405	0.106*	
C14	0.2135 (5)	0.1619 (5)	0.65781 (18)	0.0642 (11)	
H14	0.2502	0.0760	0.6515	0.077*	
C15	0.3122 (4)	0.2354 (3)	0.55912 (15)	0.0416 (8)	
C16	0.4426 (3)	0.2849 (3)	0.54164 (15)	0.0380 (7)	
C17	0.5461 (4)	0.3845 (3)	0.56847 (15)	0.0398 (8)	
C18	0.6681 (4)	0.4211 (4)	0.53799 (17)	0.0486 (9)	
H18	0.6836	0.3813	0.4999	0.058*	
C19	0.7659 (4)	0.5140 (4)	0.56250 (18)	0.0556 (10)	
H19	0.8470	0.5355	0.5411	0.067*	
C20	0.7458 (4)	0.5755 (4)	0.61809 (18)	0.0551 (10)	
H20	0.8123	0.6392	0.6344	0.066*	
C21	0.6265 (4)	0.5421 (5)	0.64936 (19)	0.0679 (12)	
H21	0.6116	0.5841	0.6871	0.082*	
C22	0.5274 (4)	0.4466 (5)	0.62564 (18)	0.0624 (11)	
H22	0.4480	0.4236	0.6480	0.075*	
Cl1	0.11229 (9)	0.69744 (9)	0.07737 (4)	0.0502 (3)	
O1	0.0560 (4)	0.5809 (4)	0.04536 (19)	0.1035 (12)	
O2	0.0600 (4)	0.8046 (4)	0.0390 (2)	0.1247 (16)	
O3	0.2657 (3)	0.6929 (4)	0.07995 (16)	0.0939 (12)	
O4	0.0591 (4)	0.7023 (5)	0.13600 (17)	0.1216 (16)	

### Atomic displacement parameters (Å^2^)

**Table:**

	*U*^11^	*U*^22^	*U*^33^	*U*^12^	*U*^13^	*U*^23^
N1	0.0325 (15)	0.0463 (17)	0.0409 (16)	−0.0013 (12)	0.0100 (12)	−0.0018 (13)
N2	0.0372 (16)	0.0509 (18)	0.0446 (16)	−0.0107 (14)	0.0112 (13)	−0.0063 (14)
C1	0.0344 (17)	0.0446 (19)	0.0403 (18)	0.0016 (14)	0.0051 (14)	−0.0009 (15)
C2	0.058 (2)	0.063 (2)	0.052 (2)	−0.0193 (19)	0.0177 (18)	−0.0104 (19)
C3	0.065 (3)	0.082 (3)	0.049 (2)	−0.011 (2)	0.0218 (19)	−0.015 (2)
C4	0.052 (2)	0.054 (2)	0.046 (2)	0.0043 (18)	−0.0002 (17)	−0.0066 (17)
C5	0.057 (2)	0.056 (2)	0.056 (2)	−0.0138 (19)	0.0006 (19)	−0.0072 (19)
C6	0.049 (2)	0.058 (2)	0.045 (2)	−0.0116 (18)	0.0103 (16)	−0.0029 (18)
C7	0.096 (4)	0.070 (3)	0.056 (3)	−0.003 (2)	0.004 (2)	−0.016 (2)
C8	0.0341 (17)	0.048 (2)	0.0406 (18)	0.0012 (15)	0.0067 (14)	0.0030 (15)
C9	0.0343 (17)	0.062 (2)	0.0396 (18)	−0.0046 (16)	0.0069 (14)	−0.0052 (17)
C10	0.055 (2)	0.065 (3)	0.060 (2)	0.0018 (19)	0.0207 (19)	−0.005 (2)
C11	0.062 (3)	0.099 (4)	0.073 (3)	0.005 (2)	0.021 (2)	−0.030 (3)
C12	0.053 (3)	0.151 (5)	0.053 (3)	−0.010 (3)	0.020 (2)	−0.034 (3)
C13	0.078 (3)	0.143 (5)	0.045 (2)	−0.012 (3)	0.017 (2)	0.018 (3)
C14	0.067 (3)	0.077 (3)	0.050 (2)	−0.001 (2)	0.013 (2)	0.008 (2)
C15	0.0412 (18)	0.0467 (19)	0.0379 (18)	−0.0004 (15)	0.0084 (14)	−0.0002 (15)
C16	0.0358 (17)	0.0428 (18)	0.0359 (17)	0.0023 (14)	0.0064 (13)	−0.0002 (14)
C17	0.0366 (17)	0.0443 (19)	0.0387 (17)	0.0017 (14)	0.0040 (14)	0.0020 (15)
C18	0.045 (2)	0.059 (2)	0.0428 (19)	−0.0067 (17)	0.0105 (16)	−0.0056 (17)
C19	0.044 (2)	0.066 (3)	0.057 (2)	−0.0101 (19)	0.0080 (18)	0.006 (2)
C20	0.050 (2)	0.057 (2)	0.057 (2)	−0.0120 (18)	−0.0031 (18)	−0.0045 (19)
C21	0.065 (3)	0.086 (3)	0.054 (2)	−0.016 (2)	0.007 (2)	−0.027 (2)
C22	0.050 (2)	0.091 (3)	0.049 (2)	−0.020 (2)	0.0150 (18)	−0.016 (2)
Cl1	0.0404 (5)	0.0565 (6)	0.0555 (6)	0.0032 (4)	0.0167 (4)	−0.0080 (4)
O1	0.093 (3)	0.089 (2)	0.130 (3)	−0.006 (2)	0.021 (2)	−0.043 (2)
O2	0.083 (3)	0.108 (3)	0.185 (4)	0.032 (2)	0.023 (3)	0.064 (3)
O3	0.0401 (16)	0.152 (3)	0.091 (2)	0.0059 (18)	0.0130 (15)	0.033 (2)
O4	0.093 (3)	0.206 (4)	0.070 (2)	−0.018 (3)	0.038 (2)	−0.037 (3)

### Geometric parameters (Å, °)

**Table:**

N1—C8	1.332 (4)	C10—H10	0.93
N1—C16	1.387 (4)	C11—C12	1.372 (7)
N1—H1	0.89 (4)	C11—H11	0.93
N2—C8	1.333 (4)	C12—C13	1.367 (8)
N2—C15	1.374 (4)	C12—H12	0.93
N2—H2	0.81 (4)	C13—C14	1.394 (6)
C1—C6	1.389 (5)	C13—H13	0.93
C1—C2	1.396 (5)	C14—H14	0.93
C1—C8	1.457 (5)	C15—C16	1.374 (4)
C2—C3	1.371 (5)	C16—C17	1.461 (5)
C2—H2A	0.93	C17—C18	1.388 (5)
C3—C4	1.370 (5)	C17—C22	1.396 (5)
C3—H3	0.93	C18—C19	1.366 (5)
C4—C5	1.378 (5)	C18—H18	0.93
C4—C7	1.509 (5)	C19—C20	1.366 (5)
C5—C6	1.370 (5)	C19—H19	0.93
C5—H5	0.93	C20—C21	1.369 (5)
C6—H6	0.93	C20—H20	0.93
C7—H7A	0.96	C21—C22	1.386 (5)
C7—H7B	0.96	C21—H21	0.93
C7—H7C	0.96	C22—H22	0.93
C9—C10	1.370 (5)	Cl1—O4	1.385 (3)
C9—C14	1.387 (5)	Cl1—O2	1.408 (4)
C9—C15	1.481 (4)	Cl1—O3	1.409 (3)
C10—C11	1.379 (5)	Cl1—O1	1.424 (3)
			
C8—N1—C16	111.2 (3)	C10—C11—H11	120.2
C8—N1—H1	120 (2)	C13—C12—C11	119.7 (4)
C16—N1—H1	127 (2)	C13—C12—H12	120.1
C8—N2—C15	110.8 (3)	C11—C12—H12	120.1
C8—N2—H2	125 (3)	C12—C13—C14	120.9 (5)
C15—N2—H2	123 (2)	C12—C13—H13	119.6
C6—C1—C2	118.1 (3)	C14—C13—H13	119.6
C6—C1—C8	121.3 (3)	C9—C14—C13	119.1 (5)
C2—C1—C8	120.5 (3)	C9—C14—H14	120.5
C3—C2—C1	120.2 (4)	C13—C14—H14	120.5
C3—C2—H2A	119.9	C16—C15—N2	106.7 (3)
C1—C2—H2A	119.9	C16—C15—C9	131.6 (3)
C4—C3—C2	121.9 (4)	N2—C15—C9	121.6 (3)
C4—C3—H3	119.0	C15—C16—N1	105.1 (3)
C2—C3—H3	119.0	C15—C16—C17	133.7 (3)
C3—C4—C5	117.6 (3)	N1—C16—C17	121.2 (3)
C3—C4—C7	120.9 (4)	C18—C17—C22	117.1 (3)
C5—C4—C7	121.5 (4)	C18—C17—C16	121.1 (3)
C6—C5—C4	122.1 (4)	C22—C17—C16	121.8 (3)
C6—C5—H5	119.0	C19—C18—C17	121.8 (3)
C4—C5—H5	119.0	C19—C18—H18	119.1
C5—C6—C1	120.1 (3)	C17—C18—H18	119.1
C5—C6—H6	120.0	C20—C19—C18	120.8 (4)
C1—C6—H6	120.0	C20—C19—H19	119.6
C4—C7—H7A	109.5	C18—C19—H19	119.6
C4—C7—H7B	109.5	C19—C20—C21	119.0 (4)
H7A—C7—H7B	109.5	C19—C20—H20	120.5
C4—C7—H7C	109.5	C21—C20—H20	120.5
H7A—C7—H7C	109.5	C20—C21—C22	121.0 (4)
H7B—C7—H7C	109.5	C20—C21—H21	119.5
N1—C8—N2	106.2 (3)	C22—C21—H21	119.5
N1—C8—C1	126.7 (3)	C21—C22—C17	120.4 (4)
N2—C8—C1	127.1 (3)	C21—C22—H22	119.8
C10—C9—C14	119.2 (3)	C17—C22—H22	119.8
C10—C9—C15	121.4 (3)	O4—Cl1—O2	112.2 (3)
C14—C9—C15	119.4 (3)	O4—Cl1—O3	112.6 (2)
C9—C10—C11	121.4 (4)	O2—Cl1—O3	110.2 (2)
C9—C10—H10	119.3	O4—Cl1—O1	109.1 (2)
C11—C10—H10	119.3	O2—Cl1—O1	103.5 (3)
C12—C11—C10	119.6 (5)	O3—Cl1—O1	108.7 (2)
C12—C11—H11	120.2		
			
C6—C1—C2—C3	−1.2 (6)	C12—C13—C14—C9	−0.8 (7)
C8—C1—C2—C3	178.9 (4)	C8—N2—C15—C16	−1.1 (4)
C1—C2—C3—C4	0.6 (7)	C8—N2—C15—C9	−179.1 (3)
C2—C3—C4—C5	0.0 (6)	C10—C9—C15—C16	66.3 (5)
C2—C3—C4—C7	−178.4 (4)	C14—C9—C15—C16	−112.8 (5)
C3—C4—C5—C6	0.0 (6)	C10—C9—C15—N2	−116.2 (4)
C7—C4—C5—C6	178.4 (4)	C14—C9—C15—N2	64.7 (5)
C4—C5—C6—C1	−0.6 (6)	N2—C15—C16—N1	0.4 (4)
C2—C1—C6—C5	1.2 (5)	C9—C15—C16—N1	178.1 (3)
C8—C1—C6—C5	−178.9 (3)	N2—C15—C16—C17	−180.0 (3)
C16—N1—C8—N2	−1.1 (4)	C9—C15—C16—C17	−2.2 (7)
C16—N1—C8—C1	179.0 (3)	C8—N1—C16—C15	0.5 (4)
C15—N2—C8—N1	1.4 (4)	C8—N1—C16—C17	−179.2 (3)
C15—N2—C8—C1	−178.7 (3)	C15—C16—C17—C18	−176.1 (4)
C6—C1—C8—N1	162.8 (3)	N1—C16—C17—C18	3.5 (5)
C2—C1—C8—N1	−17.4 (5)	C15—C16—C17—C22	4.1 (6)
C6—C1—C8—N2	−17.1 (5)	N1—C16—C17—C22	−176.2 (3)
C2—C1—C8—N2	162.7 (4)	C22—C17—C18—C19	−0.2 (6)
C14—C9—C10—C11	2.7 (6)	C16—C17—C18—C19	−179.9 (3)
C15—C9—C10—C11	−176.3 (4)	C17—C18—C19—C20	−0.7 (6)
C9—C10—C11—C12	0.6 (7)	C18—C19—C20—C21	0.5 (6)
C10—C11—C12—C13	−4.0 (7)	C19—C20—C21—C22	0.6 (7)
C11—C12—C13—C14	4.1 (7)	C20—C21—C22—C17	−1.4 (7)
C10—C9—C14—C13	−2.6 (6)	C18—C17—C22—C21	1.2 (6)
C15—C9—C14—C13	176.5 (4)	C16—C17—C22—C21	−179.1 (4)

### Hydrogen-bond geometry (Å, °)

**Table:**

*D*—H···*A*	*D*—H	H···*A*	*D*···*A*	*D*—H···*A*
N1—H1···O3^i^	0.89 (4)	2.05 (4)	2.943 (4)	175 (3)
N2—H2···O1^ii^	0.81 (4)	2.33 (4)	3.037 (5)	146 (3)
N2—H2···O1^iii^	0.81 (4)	2.50 (4)	3.196 (5)	145 (3)

Symmetry codes: (i) −*x*+1, *y*−1/2, −*z*+1/2; (ii) *x*, −*y*+1/2, *z*+1/2; (iii) −*x*, *y*−1/2, −*z*+1/2.
